# A comparison of the United Kingdom, Australian and Japanese hangover product market

**DOI:** 10.1111/dar.14030

**Published:** 2025-02-24

**Authors:** Maureen N. Zijlstra, Sanne E. Schulz, Emina Išerić, Quinten Barré, Andrew Scholey, Joris C. Verster

**Affiliations:** ^1^ Utrecht Institute for Pharmaceutical Sciences, Division of Pharmacology Utrecht University Utrecht Netherlands; ^2^ Nutrition Dietetics and Food, School of Clinical Sciences Monash University Melbourne Australia; ^3^ Centre for Mental Health and Brain Sciences Swinburne University Melbourne Australia; ^4^ Cognitive Neurophysiology, Department of Child and Adolescent Psychiatry, Faculty of Medicine Dresden Germany

**Keywords:** Australia, hangover, Japan, treatment, UK

## Abstract

**Introduction:**

The use of products to prevent or reduce alcohol hangovers is increasingly popular. The aim of this study was to evaluate and compare the alcohol hangover product markets of the United Kingdom, Australia and Japan.

**Methods:**

The website www.Amazon.com was searched, using the terms ‘hangover treatment’ and ‘hangover cure’, to identify hangover products sold in the United Kingdom, Australia and Japan. Dosage forms, ingredients and their amounts per serving were recorded and compared between the three countries.

**Results:**

The market evaluations for the United Kingdom and Australia each revealed *N* = 19 hangover products, and 24 hangover products were found for Japan. The products from the three markets were quite distinct, with none of the hangover products being marketed in all three countries. The most popular ingredients in United Kingdom were potassium (63.2%), sodium (57.9%) and vitamin C (52.6%). The most common ingredients in Australia were vitamin B1, vitamin B6, vitamin B12 and sodium (all 47.4%). In Japan, curcumin (45.8%), L‐ornithine (29.2%), vitamin C (20.8%) and vitamin B2 (20.8%) were the most popular ingredients. Most popular dosage forms also differed between the countries, with powders being most popular in the United Kingdom (42.1%), tablets in Japan (50.0%), and capsules (31.6%) and drinks (26.3%) in Australia.

**Discussion and Conclusions:**

Both ingredients and dosage forms of hangover products differed between the United Kingdom, Australia and Japan. Products also differed from the United States, illustrating the importance of cross‐cultural comparisons. As these are currently lacking, double‐blind, placebo‐controlled clinical trials are needed to demonstrate the efficacy and safety of marketed hangover products.

**Key Points**

- The hangover products marketed in the United Kingdom, Australia and Japan often comprise combinations of vitamins and minerals.
- In contrast to the United Kingdom, natural ingredients are also popular in Australia (e.g., dihydromyricetin) and Japan (e.g., curcumin).
- Most popular dosage forms of hangover products were powders (United Kingdom), tablets (Japan), and capsules and drinks (Australia).
- For none of the marketed products there is evidence from double‐blind, placebo‐controlled clinical trials in humans that the products are effective to prevent or reduce hangovers.

## 1 INTRODUCTION

Alcohol hangover is defined as the combination of negative mental and physical symptoms which can be experienced after a single episode of alcohol consumption, starting when blood alcohol concentration approaches zero [1]. Alcohol hangovers are associated with impaired cognitive and psychomotor performance and negative mood [2], and impairment of daily activities such as job performance [3] or driving a car [4]. There is ongoing discussion among researchers and policymakers about the possible risks and desirability of developing and effective hangover treatment [5]. In this context, it has been argued that such a product would increase alcohol consumption [6]. For example, a recent study in the United States found that ever‐use of hangover products among young adults was positively associated with experiencing alcohol use problems, the number of drinks consumed per typical drinking day, and the count of alcohol use disorder symptoms [6]. However, research supporting this hypothesis is limited, and a study of 1837 Dutch students found that the vast majority of them reported that the availability of an effective hangover treatment would not increase their alcohol consumption [7]. The study concluded that there is a clear need among consumers for an effective and safe hangover treatment [7]. Thus, more research is needed on the risks and benefits of the availability of effective hangover treatments.

A 2019 US market evaluation revealed that 82 hangover treatments were available via www.Amazon.com [8]. The most popular ingredients of these hangover products were vitamin B1, vitamin B6, milk thistle extract (Silymarin), dihydromyricetin (DHM) and N‐acetyl L‐cysteine (NAC). However, a literature review revealed that none of the marketed hangover products had been evaluated for safety or efficacy via double‐blind, placebo‐controlled studies in humans [9].

Hangover products, their ingredients and dosage forms may differ between countries and continents. Previously, we evaluated the United States [8]. Therefore, the aim of the current study was to identify the available hangover products in the United Kingdom, Japan and Australia, and to compare the ingredients and dosage forms of these hangover products between the countries. The countries were selected because they each have a considerable hangover product market size, but are from three different continents. Australia and the United Kingdom have the biggest market share of their corresponding continents (Australia and Europe, respectively), whereas Japan is one of the fastest growing hangover product markets in Asia [10]. However, the countries have clearly distinctive cultures. This is evident for popularity of different alcoholic beverage types, the quantity of alcohol consumed, food preferences and ingredients and dosage forms of medicines and supplements that are used [11, 12, 13]. It is hypothesised that these country‐specific characteristics are reflected in differences in ingredients of hangover products that are available in these countries.

## 2 METHODS

The website www.Amazon.com was searched, using the terms ‘hangover treatment’ and ‘hangover cure’, to identify hangover products sold in the United Kingdom (1 October 2023), Australia (13 January 2024) and Japan (23 November 2024). The delivery location was set at London, Sydney and Tokyo, respectively. The search resulted in 390 hits for the United Kingdom, 454 for Australia and 325 hits for Japan. Duplicates were removed. Products were not considered if there was no reference to the alcohol hangover, or if the product's use was unrelated to alcohol consumption. Finally, hangover products without active ingredients (e.g., facemasks and cooling eye pads) were excluded. Of the remaining products, information on the dosage form, ingredients and their dosage per serving were collected from the images and product description on the product's Amazon website. Only ‘active’ ingredients were listed (i.e., those on the nutrition label or part of the ‘proprietary blend’). Inactive ingredients (e.g., preservatives and fillers) were not considered.

Concerning dosage safety, the number of products was determined that included an amount of ingredient per serving that exceeded the recommended dietary intake (RDI) level or the upper level of intake (UL), that is, the highest nutrient intake level likely to pose no adverse health effects. The RDI and UL levels for the United Kingdom were collected from the National Health Service [14] and the British Nutrition Foundation websites [15]. For Australia, the RDI and UL levels were obtained from the National Health and Medical Research Council [16]. For most Japanese hangover products, the dosage of the ingredients was not disclosed on the product, package or Amazon website text. Therefore, no analyses could be conducted on dosage levels of the hangover products sold in Japan, or whether these dosages exceeded the RDI or UL.

## 3 RESULTS

The market evaluation for the United Kingdom revealed *N* = 19 hangover products. The different dosage forms included powders (8), tablets (4), capsules (3), transdermal patches (2), and drinks and liquids (2). The 20 most popular ingredients of these products are listed in Table 1. These included potassium (12 products), sodium (11) and vitamin C (10). For seven vitamins, one or more product exceeded the RDI, and one hangover product exceeded the UL for vitamin C. Compared with the United States [8], ‘natural’ ingredients were relatively uncommon in UK hangover products: Beetroot and milk thistle were included in 3 products (15.8%) and 2 products (10.5%), respectively, whereas DHM was not included in any of the 19 hangover products.

**TABLE 1:** The 20 most common ingredients of hangover products marketed in the United Kingdom.

	Ingredient	Number of products	Dose not specified	Dose range	Products exceeding RDI	Products exceeding UL
1	Potassium	12 (63.2%)	1	99–578 mg	0 (0%)	0 (0%)
2	Sodium	11 (57.9%)	4	75–500 mg	0 (0%)	ND
3	Vitamin C	10 (52.6%)	1	25–1200 mg	7 (77.8%)	1 (11.1%)
4	Vitamin B12	9 (47.4%)	1	1.25–25 μg	7 (87.5%)	0 (0%)
5	Vitamin B1	8 (42.1%)	2	0.45–5 mg	3 (50%)	ND
6	Vitamin B3	7 (36.8%)	1	5–27 mg	2 (33.3%)	0 (0%)
7	Magnesium	6 (31.6%)	1	56–109 mg	0 (0%)	0 (0%)
8	Zinc	6 (31.6%)	0	1.5–4 mg	0 (0%)	0 (0%)
9	Vitamin B5	5 (26.3%)	0	3–50 mg	ND	0 (0%)
10	Vitamin B9	5 (26.3%)	1	33–250 μg	1 (25%)	0 (0%)
11	Vitamin B6	5 (26.3%)	0	1.17–10 mg	4 (80%)	0 (0%)
12	Vitamin B2	4 (21.1%)	0	1.17–5 mg	0 (0%)	ND
13	Beetroot	3 (15.8%)	3	ND	ND	ND
14	Selenium	3 (15.8%)	0	17–50 μg	0 (0%)	0 (0%)
15	Vitamin E	3 (15.8%)	1	1.8–10 mg	1 (50%)	0 (0%)
16	Activated charcoal	2 (10.5%)	2	ND	ND	ND
17	L‐ascorbic acid	2 (10.5%)	2	ND	ND	ND
18	Milk thistle	2 (10.5%)	2	ND	ND	ND
19	NAC	2 (10.5%)	1	600 mg	ND	ND
20	Peppermint	2 (10.5%)	1	2066 mg	ND	ND

Abbreviations: NAC, N‐acetyl‐cysteine; ND, not determined, RDI, recommended dietary intake; UL upper level of intake.

The market evaluation for Australia also revealed *N* = 19 hangover products. Dosage forms included capsules (6), drinks and liquids (5), powders (4), transdermal patches (3), and tablets (1). The 20 most popular ingredients of Australian hangover products are listed in Table 2. The most common ingredients in Australia were vitamin B1 (9 products), vitamin B6 (9), vitamin B12 (9) and sodium (9). For nine vitamins, one or more product exceeded the RDI, and one hangover product exceeded the UL for vitamin B9. Natural ingredients were more popular in Australia compared to the United Kingdom. In Australia, popular natural ingredients included DHM (seven products, 36.8%), ginger extract (five products, 26.3%) and milk thistle (five products, 26.3%).

**TABLE 2:** The 20 most common ingredients of hangover products marketed in Australia.

	Ingredients	Number of products	Dose not specified	Dose range	Products exceeding RDI	Products exceeding UL
1	Vitamin B1	9 (47.4%)	1	1.2–150 mg	7 (87.5%)	ND
2	Vitamin B6	9 (47.4%)	0	1.7–29 mg	8 (88.9%)	0 (0%)
3	Vitamin B12	9 (47.4%)	0	2.4–1000 μg	7 (77.8%)	ND
4	Sodium	9 (47.4%)	0	5–410 mg	0 (0%)	ND
5	Vitamin C	8 (42.1%)	1	22.5–1000 mg	5 (71.4%)	ND
6	Potassium	8 (42.1%)	0	15–160 mg	0 (0%)	ND
7	DHM^a^	7 (36.8%)	3	300–960 mg	ND	ND
8	Vitamin B2	7 (36.8%)	1	0.08–10 mg	4 (66.7%)	ND
9	Vitamin B3	6 (31.6%)	1	2.3–50 mg	3 (60.0%)	0 (0%)
10	Vitamin B5	6 (31.6%)	1	5–15 mg	2 (40.0%)	ND
11	Ginger extract	5 (26.3%)		50–1000 mg	ND	ND
12	Milk thistle	5 (26.3%)	1	50–250 mg	ND	ND
13	Magnesium	4 (21.1%)	1	9–18 mg	0 (0%)	0 (0%)
14	Vitamin B7	3 (15.8%)	1	15–300 μg	1 (50.0%)	ND
15	Vitamin B9	3 (15.8%)	1	1.3–4000 μg	1 (50.0%)	1 (5.3%)
16	Vitamin E	3 (15.8%)	0	2.5–10 mg	0 (0%)	0 (0%)
17	Calcium	3 (15.8%)	0	3–55 mg	0 (0%)	0 (0%)
18	Zinc	3 (15.8%)	0	0.2–10 mg	0 (0%)	0 (0%)
19	NAC	2 (10.5%)	1	300 mg	ND	ND
20	Alpha lipoic acid	2 (10.5%)	1	250 mg	ND	ND

Abbreviations: DHM, Dihydromyricetin; NAC, N‐acetyl‐cysteine; ND, not determined, RDI, recommended dietary intake; UL upper level of intake.

^a^ Also includes DHM from Japanese raisin tree and hovenia dulcis.

The market evaluation for Japan revealed *N* = 24 hangover products. Most popular dosage forms were tablets (12 products) and capsules (5), followed by powders (3), granules (2), soft chews (1) and gel/jelly (1). The 20 most popular ingredients of Japanese hangover products are listed in Table 3. For one product, no ingredients were reported and for some other products the disclosure of ingredients was incomplete (e.g., it was stated that the product included a blend of unspecified vitamins, minerals or essential amino acids). In contrast to the United Kingdom and Australia, Japanese hangover products and their package usually did not have a nutrition label. The most commonly reported ingredients in Japan were curcumin (11 products), L‐ornithine (7), vitamin C (5) and vitamin B2 (5). No analysis could be conducted on dosage levels of the hangover products sold in Japan.

**TABLE 3:** The 20 most common ingredients of hangover products marketed in Japan.

Ranking	Ingredient	Number of products (%)
1	Turmeric, curcumin	11 (45.8)
2	L‐Ornithine	7 (29.2)
3	Vitamin C	5 (20.8)
4	Vitamin B2	5 (20.8)
5	Vitamin B1	4 (16.7)
6	Niacin	4 (16.7)
7	Zinc	4 (16.7)
8	Vitamin B12	3 (12.5)
9	Milk thistle	3 (12.5)
10	Alanine	3 (12.5)
11	Hepa (liver extract)	3 (12.5)
12	Clam extract	2 (8.3)
13	Vitamin E	2 (8.3)
14	Vitamin B6	2 (8.3)
15	Vitamin B9	2 (8.3)
16	Guava	2 (8.3)
17	Arginine	2 (8.3)
18	Calcium	2 (8.3)
19	Iron	2 (8.3)
20	Mangelicon	2 (8.3)

## 4 DISCUSSION

The hangover product markets in United Kingdom, Australia and Japan are very different. None of the hangover products were marketed in all three countries, and only two products were available in both the United Kingdom and Australia. While the top 12 ingredients in the United Kingdom comprised of only vitamins and minerals, the Australian hangover products also included ‘natural’ ingredients such as DHM, ginger extract and milk thistle. In Japan, curcumin and L‐ornithine were the most common ingredients. As such, the Australian hangover product market is more comparable to the United States [8] than the United Kingdom. In both the United Kingdom and Australia, NAC was much less common as ingredient compared to the United States. In Japan, NAC was not used as ingredient. Alternatively, while curcumin and L‐ornithine were most popular in Japan, they were not included in the top 20 of ingredients of the United Kingdom and Australia. Regarding dosage forms, in the United Kingdom powders were most popular, tablets in Japan, whereas in Australia capsules and drinks were most popular. Hangover products without active ingredients and those that do not need to be ingested by consumers (e.g., facemasks, cooling eye pads, wristbands and drinking glass purifiers) were excluded from the current market evaluations. However, in contrast to Australia and Japan, these hangover products were also popular in the United Kingdom.

The differences in popular ingredients and dosage forms of hangover products might reflect cultural differences between the countries. These cultural differences may include attitudes towards ‘natural’ ingredients and public acceptance of complementary medicine. In the United Kingdom, natural ingredients and herbal medicine are primarily used and appreciated by ethnic groups [11], whereas in Australia the use of complementary medicine products is much more accepted and practised by the general population as a whole [12]. The analysis also revealed differences in most common delivery formats of the products. Differences between countries in preferences for delivery forms have been previously reported [13]. In addition to this, different dietary product regulations between the countries may have an influence on the observed differences in ingredients and modes of serving.

A literature search revealed no randomised double‐blind, placebo‐controlled clinical trials that demonstrated the safety and efficacy of any of the hangover products marketed in the United Kingdom, Australia and Japan. Regarding individual ingredients, except for 1 study showing positive results for vitamin B6 [17], there are no clinical trials conducted in humans demonstrating that vitamins or minerals can prevent or reduce hangovers. Regarding natural ingredients, a double‐blind, placebo‐controlled study revealed that DHM did not reduce hangover severity [18].

When developing a hangover product, companies may combine ingredients and market them for preventing or reducing the alcohol hangover. However, besides the fact that there is very limited evidence of any efficacy of these ingredients [9], this practise raises health safety concerns. While ingredients may be generally recognised as safe while considered individually, it is unknown whether unwanted interactions occur in combination products. This may be of particular concern for natural ingredients. Also, DRI and UL levels for natural ingredients have not been determined, and it is unknown whether these compounds interact with prescription drugs consumers may use. In addition to harmful interactions, there is also a risk of overconsumption of hangover products which may result in unwanted side effects. Therefore, in the interest of consumers, it is important that companies conduct clinical trials to demonstrate the efficacy and safety of their hangover product. Regulatory agencies should also address these potential safety risks of hangover products that are not properly evaluated.

To improve future comparisons of hangover products between countries it is essential that all ingredients and the dosages per serving size are mentioned on the product nutrition label. Preferably, product formulations and standardised labels will be harmonised across countries.

It is warranted to continue monitoring the hangover product markets in the United Kingdom, Australia and Japan. For this purpose, the Amazon website was used. This can be viewed as a limitation, as hangover products can also be bought at other websites, supermarkets, pharmacies, and drinking venues. However, Amazon is the biggest online platform in the United Kingdom, Australia and Japan, and it is therefore reasonable to assume that searching this online platform provides a reliable overview of the hangover product markets of the three countries.

In conclusion, the hangover products marketed in the United Kingdom, Australia and Japan often comprise combinations of vitamins and minerals. However, in contrast to products sold in the United Kingdom, natural ingredients such as DHM and ginger extract are also popular ingredients of hangover products sold in Australia. In Japan, curcumin and L‐ornithine are most popular ingredients. Of concern, for none of the marketed products there is evidence from double‐blind, placebo‐controlled clinical trials in humans that the products are effective or safe.

## AUTHOR CONTRIBUTIONS

Conceptualisation: Joris C. Verster, Maureen N. Zijlstra, Quinten Barré, Sanne E. Schulz, Emina Išerić and Andrew Scholey; writing – original draft preparation: Maureen N. Zijlstra and Joris C. Verster; data collection: Maureen N. Zijlstra and Sanne E. Schulz; data curation and analysis: Maureen N. Zijlstra, Quinten Barré, Sanne E. Schulz and Emina Išerić; writing – review and editing: Joris C. Verster, Maureen N. Zijlstra, Quinten Barré, Sanne E. Schulz, Emina Išerić and Andrew Scholey. All authors have read and agreed to the published version of the manuscript.

## CONFLICT OF INTEREST STATEMENT

Over the past 36 months, Joris C. Verster has acted as a consultant/expert advisor to Eisai, KNMP, Med Solutions, Mozand, Red Bull, Sen‐Jam Pharmaceutical and Toast!. Joris C. Verster has received travel support from Sen‐Jam Pharmaceutical and owns stock from Sen‐Jam Pharmaceutical. Andrew Scholey has acted as a consultant/expert advisor to Bayer, Coca Cola, Danone, Delica Therapeutics, GlaxoSmithKline, Givaudin, Liquid IV, Mars‐Wrigley, Naturex, Nestlé, McCormick, Metavate Consultancy, PepsiCo, Pfizer, Pharmavite, REVIV, Sanofi, Verdure Sciences and Wörwag Pharma; and in the past 36 months Scholey has held research grants from Abbott Nutrition, Arla Foods, the Australian Research Council, Bayer, BioRevive, DuPont, Fonterra, GlaxoSmithKline, the High Value Nutrition Fund, the National Health and Medical Research Council, Nutricia‐Danone, Sanofi and Wörwag Pharma. Andrew Scholey is Chief Scientific Officer for Ārepa Nootropics and is on the Scientific Advisory Board of Sen‐Jam Pharmaceutical. He has stock from Sen‐Jam Pharmaceutical and Ārepa Nootropics. He has received travel support from Vitafoods and ILSI. Maureen N. Zijlstra and Emina Išerić have received travel support from Sen‐Jam Pharmaceutical. The other authors have nothing to declare.

## Data Availability

The data that support the findings of this study are available from the corresponding author upon reasonable request.
